# Tuina for lumbar disc herniation: A protocol for systematic review and meta analysis

**DOI:** 10.1097/MD.0000000000024203

**Published:** 2021-01-08

**Authors:** Zuoxiong Miao, Zhenglan Tong, Jinfei Ye, Shihan Leng, Min Wang, Anli Hu, Jingyu Zhang, Xingze Dai, Jiarong Liang, Yunlong Geng, Liang Chen, Bin Ye, Youkang Dong

**Affiliations:** a School of Acupuncture-Tuina and Rehabilitation, Yunnan University of Chinese Medicine, Kunming; b The first Affiliated Hospital of Guangzhou University of Chinese Medicine, Guangzhou, China.; c Clinical Medical college, Yunnan University of Chinese Medicine; d Yunnan St.John's Hospital; e The first Affiliated Hospital, Yunnan University of Chinese Medicine/Yunnan Provincial Hospital of Traditional Chinese Medicine, Kunming, China.

**Keywords:** Lumbar disc herniation, systematic review protocol, tuina

## Abstract

**Background:**

Lumbar disc herniation (LDH) is an important factor of causing leg pain and numbness. As a secondary discipline of Traditional Chinese Medicine, tuina is widely used for the treatment of LDH in China even in other nations while its clinical value is not acknowledged universally. So, we focus on this article aims to evaluate its efficacy and safety of LDH.

**Methods:**

Electronic databases involving Cochrane Library, PubMed, Web of Science, EMBASE, China Science and Technology Journal, China National Knowledge Infrastructure, Wanfang and Chinese Biomedical Literature Database will be pertained with appropriate search strategy. And RevMan V.5.3.5 software will be conducted as the assessment tool for bias risk, data synthesis, subgroup analysis as well as meta-analyses.

**Results:**

This systematic review will provide a high-quality synthesis of current evidence of tuina for LDH.

**Conclusion:**

This protocol will determine whether Tuina is an effective and safe treatment method for LDH.

## 1 Introduction

Lumbar disc herniation (LDH) refers to a series of symptoms and signs caused by oppression and irritation to nerve root due to fibrous ring rupture and nucleus pulposus herniation. It is an age-related degenerative disease and characteristics by a rapid onset, long disease duration, difficult to achieve satisfied effectiveness and easy to relapse in a short period of time, posing critical challenges to physical and mental health of patients.^[1,2]^ Up to now, The prevalence of LDH in the general population is about 2% to 5%,^[3]^ which brings heavy economic burden to the family and society.^[4]^ LDH belongs to *back pain* or *back and leg pain* in Traditional Chinese Medicine, and with its etiology and pathogenesis including accidental injury, excessive labor intensity, deficiency of liver and kidney,blood stasis, invasion of pathogenic wind, cold and dampness and so on.^[5]^

Clinically, conservative methods involving tuina, acupuncture, physical therapy, moxibustion, Chinese herbs have been generally using and achieving certain clinical effects in the treatment of LDH.^[6–8]^ Among them, as the main method, tuina has been accepting by majority patients attributes to its good advantages of low risk, low cost, and good effectiveness.

According to the latest study,^[9]^ Tuina provides statistical significant benefits in the treatment of LDH by means of relaxing the tense muscles, loosening the adhesions, improving the blood circulation and the absorption of local inflammation and edema contributed to the restoration of nerve function as well as other soft tissues. Although a myriad of randomized controlled(clinical) trials (RCTs) have been supported its value of treatment,^[10]^ the conclusions are still uncertain owing to differences in operator methods, proficiency, treatment experience, and selection of treatment sites.

In this review, by retrieval, extraction and analysis of relative literatures, we intend to assess the efficacy and safety of tuina on LDH contributing to clinical therapy.

## 2 Methods

This systematic review protocol has been registered on PROSPERO with the number of CRD42019126400, and was performed in accordance with the Preferred Reporting Items for Systematic Reviews and Meta-analysis Protocol (PRISMA-P).^[11]^ This is a literature based study, so ethical approval is not necessary.

### 2.1 Selection criteria

#### 2.1.1 Types of study

All RCTs of Chinese and English literatures in the treatment of LDH will be included with publication status restrictions.

#### 2.1.2 Types of patients

We will include patients between the ages of 18 and 70 who have been diagnosed with LDH by computed tomography(CT) or Magnetic Resonance Imaging in the past 10 years. However, studies with specific or systemic diseases (such as hematopathy, spinal tumor, caudal equina syndrome, Lumbar spondylolisthesis, fracture, severe osteoporosis, and pregnancy patients will be excluded.

#### 2.1.3 Types of interventions

The experimental group was treated with tuina while the control 1 was treated with approved methods such as oral medicine, physical therapy, behavioral therapy or acupoint therapy, and so on.

### 2.2 Outcome measures

The primary evaluation indicator will be the visual analog scale,^[12]^ and the secondary outcome will include the short-form 36(SF-36)item health survey questionnaire.

### 2.3 Data sources

The main data sources of this study include electronic resource database, trial registries, retroactive references and grey literature. So we will retrieve and extract documents from Cochrane Library, PubMed, Web of Science, EMBASE, China Science and Technology Journal,China National Knowledge Infrastructure, Wanfang and Chinese Biomedical Literature Database and other electronic databases in both Chinese and English electronic databases from the publication to February, 2020.

### 2.4 Search strategy

The search strategy will be based on the guidance of the Cochrane Handbook including Medical Subject Heading terms and variants: “lumbar disc herniation,” “Low back pain,” “sciatica,” “Back Pain,” “herniated disk,” “disc prolapse,” “lumbago,” “Lumbar disc protrusion,” “tuina,” “Chinese tuina,” “massage,” “Chinese massage,” “therapy,” “manual therapy,” “Chinese manipulation,” “Chinese manipulative therapy,” “Massotherapy,” “Acupressure,” and all possible spellings of “lumbar disc herniation” and “tuina.” The search strategy is listed in Table 1, Literatures that do not meet the inclusion criteria will be excluded. All different points of view will be solved by discussion.

**Table 1:** Web of science search strategy.

Number	Search terms
1	Randomized controlled trial
2	Randomized clinical trial
3	Controlled clinical trial
4	Randomly
5	Randomized
6	Trial
7	Or/1–6
8	Lumbar disc herniation
9	Lumbar disc protrusion
10	Disc prolapse
11	Herniated disk
12	Low back pain
13	Lumbago
14	Back pain
15	Sciatica
16	Or/8-15
17	Tuina
18	Chinese tuina
19	Massage
20	Chinese massage
21	Chinese manipulation
22	Chinese manipulative therapy
23	Manual therapy
24	Massotherapy
25	Therapy
26	Acupressure
27	Or/17-26
28	7,16 and 27

### 2.5 Study selection

**Figure 1:**
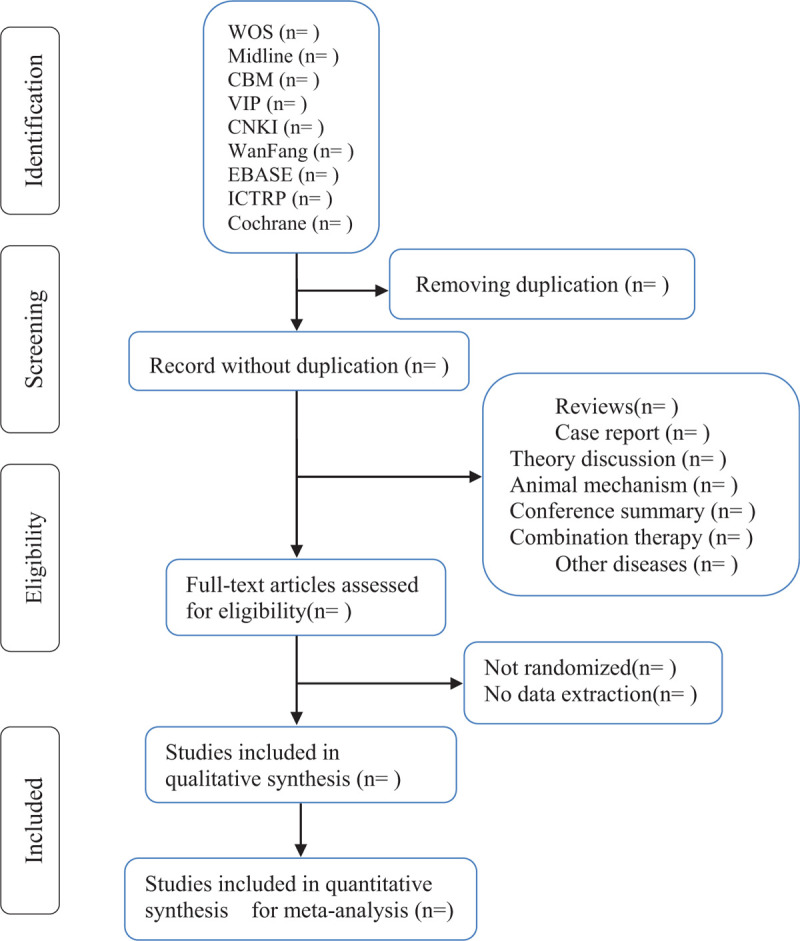
Flow diagram of studies identified.

Two researchers will search the key words, abstracts and titles of qualified references independently. Finally, they will decide which trials meet the inclusion criteria. If there is any disagreement, they will discuss and contact with the authors to understand the relevant research. The research summary of the screening flow chart is shown in Fig. 1.

### 2.6 Data extraction

Using the electronic form, Jinfei Ye and Min Wang will extract the substantive content of each article respectively. The information should include: the first author or corresponding author, publication time, design of study which contains blinding, assignment concealment, randomization and case report, inclusion and exclusion criteria, gender, age, courses, treatment process, prognosis, follow-up, even if the occurrence unexpected side effects, and so on. The third reviewer, Shihan Leng, will review the data again. We will call on the author to actively integrate the research information in case of information loss. The differences will be resolved after consultation with experts and arbitrators.

### 2.7 Quality assessment

According to Cochrane handbook for systematic reviews of interventions, Jinfei Ye, and Zuoxiong Miao will respectively conduct biased risk assessment from 7 domains: personnel and outcome, selective reporting, allocation concealment, random sequence generation, blinding, incomplete outcome data and other issues. In addition, these areas will be divided into 3 categories according to low risk of bias, high risk of bias as well as ambiguous risk. The 2 reviewers will evaluate the results using Grading of Recommendations Assessment, Development and Evaluation.^[13,14]^ All different points of view will be discussed and agreed upon.

### 2.8 Dealing with missing data

If we encounter missing data, we will contact the author and try to obtain the data as soon as possible. If data is not available, that study will not be included in the data analysis.

### 2.9 Assessment of heterogeneity and data synthesis

We will use Cochrane Collaboration's Revman 5.3 software for meta-analysis. Q-test and I^2^ statistics will be used to evaluate the heterogeneity of the data. When *I*^2^ is over 25%, 50%, and 75%, respectively, it indicated that there will be low, medium and high heterogeneity among the studies. When I^2^ is greater than or equal to 50%, it means substantial heterogeneity. If *I*^2^ is less than 50%, the fixed effect model will be conducted for analysis. If *I*^2^ is greater than or equal to 25%, the pooled data of age, sex, race, kinds of tuina, sample size and other factors will be used by the random effect model. In addition, in order to explore the causes of heterogeneity, we will consider and complete subgroup analysis, regression analysis and data-based sensitivity analysis.

### 2.10 Assessment of reporting bias

When the trials in the meta-analysis are greater than or equal to 10, we will create a funnel plot to assess the bias of the report.

### 2.11 Subgroup analysis

If we find substantial heterogeneity in the included studies, subgroup analysis will be conducted according to race, age, sex, kinds of tuina, sample size and other factors.

### 2.12 Sensitivity analysis

In this study, if there are enough trials data, sensitivity analysis will be conducted based on sample size, statistical model, heterogeneity qualities to determine whether the conclusion is robust.

### 2.13 Ethics and dissemination

The protocol does not need to be approved by ethics, in addition, we will publish the results in peer-reviewed journals.

## 3 Discussion

LDH is caused by accumulated injuries on the basis of degenerative changes, and is a common and frequently occurring disease in tuina, acupuncture, rehabilitation orthopedics, nerve as well as other clinical departments. With the change of lifestyle and work pattern, sedentary phenomenon is more prominent among individuals, whom pay no attention to the height of chair, posture and sedentary time. So, the incidence of LDH is increasing gradually showing various stage of age, bringing serious burden on individuals, families and society financially.^[15]^

As a main therapy, tuina plays a significant role in treatment of LDH by means of relaxing spasmodic muscles in lumbar back region, improving the abnormal anatomical position of the lumbar spine, alleviating the symptoms of nerve compression and the pressure behind the lumbar disc, increasing the internal stability of the spine. It has been showing great curative effect in the improvement of symptoms,signs and pain scores, etc. Unfortunately, there is no systematic review or meta-analysis of its safety and effectiveness has been published and evaluated systematically and scientifically yet.

To sum up, it might be essential to make a systematic review and meta-analysis based on the RCTs by using the method of EBM to estimate tuina for LDH, and to provide a strong evidence of application of tuina for LHD safely and effectively.

## Author contributions

**Conceptualization**: Youkang Dong.

**Data curation:** Jinfei Ye, Min Wang, Shihan Leng.

**Formal analysis**: Zuoxiong Miao.

**Funding acquisition:** Bin Ye, Youkang Dong.

**Project administration**: Bin Ye.

**Resources:** Bin Ye, Youkang Dong.

**Writing – original draft:** Zuoxiong Miao, Zhenglan Tong, Jinfei Ye, Shihan Leng.

**Writing – review & editing:** Zuoxiong Miao, Zhenglan Tong.
